# Mapping quantitative trait loci underlying body weight changes that act at different times during high‐fat diet challenge in collaborative cross mice

**DOI:** 10.1002/ame2.70144

**Published:** 2026-03-06

**Authors:** Hanifa J. Abu‐Toamih Atamni, Iqbal M. Lone, Ilona Binenbaum, Kareem Midlej, Eleftherios Pilalis, Richard Mott, Aristotelis Chatziioannou, Fuad A. Iraqi

**Affiliations:** ^1^ Department of Clinical Microbiology and Immunology, Faculty of Medicine and Health Sciences Tel‐Aviv University Tel‐Aviv Israel; ^2^ Center of Systems Biology Biomedical Research Foundation of the Academy of Athens Athens Greece; ^3^ Division of Pediatric Hematology‐Oncology, First Department of Pediatrics National and Kapodistrian University of Athens Athens Greece; ^4^ e‐NIOS Applications PC Kallithea Greece; ^5^ Department of Genetics University College of London London UK

**Keywords:** candidate genes, collaborative cross mice, high‐fat diet, obesity, QTL mapping

## Abstract

Over one billion people worldwide suffer from obesity, and the number is continually rising. This epidemic is partly caused by the modern lifestyle. Animal models, especially mouse models, are crucial to identifying the genetic components of complex disorders and exploring the potential applications of these genetic findings. The body weight of the animals used in research is often measured regularly to monitor their health. Only endpoint measurements, such as ultimate body weight, are frequently examined in quantitative trait locus (QTL) studies; time series data, including weekly or biweekly body weight, are usually disregarded. QTL mapping using biweekly body weight measurements may be particularly intriguing in examining body weight gain in obesity research and identifying more genes associated with obesity and related metabolic disorders. This study is focused on identifying quantitative trait loci (QTLs) underlying body weight changes by analyzing biweekly weight measurements in collaborative cross (CC) mice maintained on a high‐fat diet for 12 weeks. QTL analysis, utilizing 525 mice from 55 CC lines (308 males and 217 females), revealed genome‐wide significant QTLs on different chromosomes for body weight changes over 12 weeks. This study unveiled 62 body weight QTLs, among which 28 novel QTLs associated with defined traits were observed and found not reported previously. In addition, 34 more QTLs were fine‐mapped, as the genomic interval positions of these had been previously identified. These findings highlight genomic regions that influence body weight in CC mice, underscoring the value of time series data in identifying novel genetic factors.

## Graphical abstract

Over one billion people worldwide suffer from obesity, and the number is continually rising. Animal models, especially mouse models, are crucial to identifying the genetic components of complex disorders and exploring the potential applications of these genetic findings. Only endpoint measurements, like ultimate body weight, are frequently examined in quantitative trait locus (QTL) studies; time series data, such as weekly or biweekly body weight, are usually disregarded. This study is focused on identifying quantitative trait loci (QTLs) underlying body weight changes by analyzing biweekly weight measurements in collaborative cross (CC) mice maintained on a high‐fat diet for 12 weeks. QTL analysis, utilizing 525 mice from 55 CC lines (308 males and 217 females), revealed genome‐wide significant QTLs on different chromosomes for body weight changes over 12 weeks. This study unveiled 62 body weight QTLs, among which 28 novels associated with defined traits were observed and found not reported previously. In addition, 34 more QTLs were fine‐mapped as the genomic interval positions of these were previously identified.

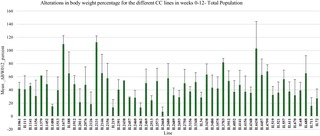

## 1 INTRODUCTION

The pathophysiology of obesity is known to be influenced by both inherited and environmental factors, and it differs significantly across populations and ethnic groups. Environmental influences, including those that may or may not contribute to obesity, can only be predicted by the host's genetic composition. Several genetic alterations induce a phenotypic alteration only under specific environmental conditions. Finding additional QTL for a more accurate analysis of obesity thus becomes a question in the QTL analysis of this condition. Consequently, to be included in this study, a search was conducted for unique body weight–specific QTL mapping in collaborative cross (CC) mice. Diseases related to gene–environmental interactions are commonly thought to be influenced by complex factors, including numerous host genes and obesity. According to current thinking, dietary decisions influence how genes are expressed, but genetics also plays a significant role in the development of one or more features of metabolic syndrome. This investigation focuses on the genes associated with the identified QTL in the subsequent stage.

More than one billion people in the world are obese, and the incidence is further increasing.^1^, ^2^ However, the stage is set by genetic variation, which influences the development and progress of the disease. Animal models, particularly mouse models, are crucial for elucidating the genetic contributions to complex diseases and for further investigating the function of these genetic discoveries. In most QTL investigations, only data such as final body weight as endpoint measurements are studied; time series data, such as weekly or biweekly body weight, are typically ignored. To investigate body weight gain in obesity research and identify additional genes associated with obesity and related metabolic disorders that exhibit their effect more prominently during development or disease progression and may fade or disappear later, QTL mapping using biweekly body weight measurements may be particularly interesting. The next‐generation mouse genetic reference population (GRP), known as the CC mice, is based on the use of various wild‐derived strains and outbred mice. It is believed that the wild‐derived strains contributed to the enrichment of genetic diversity during the generation of the CC lines.^3^, ^4^, ^5^

Since the development of genome‐wide association studies (GWAS), the identification of obesity susceptibility variants has improved. Understanding the etiology of such complicated characteristic disorders as obesity and metabolic syndrome, therefore, requires identifying genetic markers for these conditions in populations with similar environmental exposure. In the current report, we fine‐mapped previously identified QTL associated with body weight increase. We went on to show how to use extensive phenotypic data on body weight development assessment to identify new QTL and genes associated with body weight change in response to a high‐fat diet (HFD). In the current study, we focused on biweekly visits in this CC population from week 12 to week 20. These data allowed us to identify body weight QTL that contributes to the overall obese phenotype peculiar to the CC lines and the known major QTL on different chromosomes.

## 2 MATERIALS AND METHODS

### 2.1 Ethics statement

According to the standard protocol authorized by the Animal Use and Care Committee at Tel Aviv University (TAU), mice were housed in the Faculty of Medicine's small animal facility (approved experiment number M‐12‐025).

### 2.2 CC lines, animal housing, and diet

The mice were housed in open‐top cages with hardwood chip bedding, separated by gender and CC line, and maintained under a 12 h light/dark cycle. Table S1 displays the specifics of the mice employed and the distribution of all the CC lines in each sex.

### 2.3 Genotyping and phenotyping

All CC lines were genotyped with a high‐density custom‐designed Mouse Universal Genotyping Array (MegaMUGA)—built on an Illumina platform consisting of approximately 230 000 single nucleotide polymorphisms (SNPs), which are available at http://mtweb.cs.ucl.ac.uk/mus/www/preCC/MEGA_MUGA/Mar2015.MEGA+MDA+MUGA/.^6^

### 2.4 QTL mapping

Data analysis was performed using the statistical software R, including the HAPPY package.^6^ Briefly, the HAPPY package also tests for the existence of a QTL at each locus using the estimated probabilities of descent from the founder strains to estimate the phenotypic effect attributable to each founder strain. If these effects are significantly different, then there is evidence for QTL. Thus, for the initial stage of linkage analysis based on single‐trait analysis, a standard polyallelic “marker” model will be used. This model tests to determine whether there is an overall association between the marker and the trait by comparing the results after fitting the complete model, which includes the mean, any fixed effects such as generation, and the marker alleles. Initially, we performed 1000 permutation tests to determine the statistical genome‐wide significance thresholds of −log10 (*p*)‐values corresponding to 95%, 90%, and 50% of the marker‐based heritability of the trait. Subsequently, the 95%, 90%, and 50% confidence intervals (CIs), which are above the threshold values, provided the genomic intervals in which the marker associated with the assessed trait is likely to be mapped.

### 2.5 Founder's effect analysis and statistical analysis

Sequence variations that segregate in the CC should segregate in the CC founders as well, except for a few de novo mutations that arise during breeding. We examined which variations matched the pattern of activity at the QTL under QTL peak.^6^

### 2.6 Gene prioritization and functional analysis

The Mouse Genome Informatics (MGI) database resource, available at https://www.informatics.jax.org/, was utilized for the functional interpretation and gene and cellular pathway prioritization analysis of the QTL results.^7^

## 3 RESULTS

After 12 weeks of the HFD challenge, the obesity effect was assessed to determine the response of the CC lines. Using the HAPPY software, the genotypic data of the CC lines were analyzed to determine any genetic correlation with the body weight features for both the male and female populations and the entire population.

### 3.1 Significant body weight variation between CC lines

**FIGURE 1:**
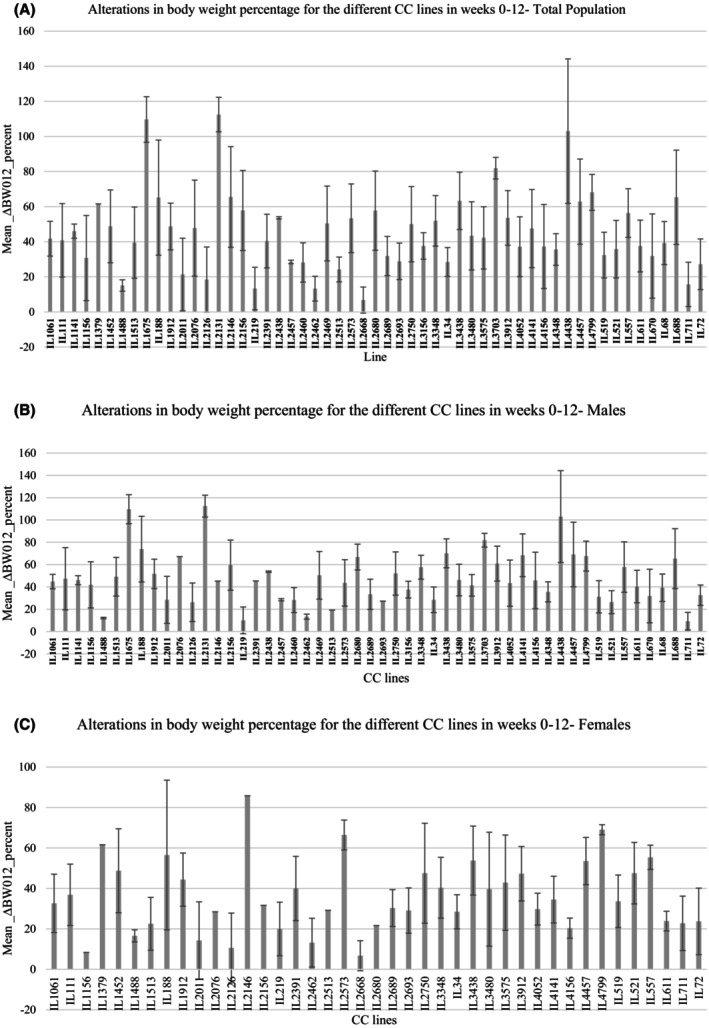
Total time series data of body weight at different time points of the mice age of different collaborative cross (CC) lines in response to high‐fat diet (HFD, 42%) challenge. Bar graph (A) shows the body weight of total time series data of 55 CC lines of the overall CC lines. Bar graph (B) shows the body weight of total time series data of females from 41 CC lines. Bar graph (C) shows the body weight of total time series data of males from 52 CC lines. The *X*‐axis represents the line number of different CC lines. The *Y*‐axis represents the total time series body weight data (min × mg/dL).

Figure 1A–C illustrates the significant differences in percentage body weight means between the entire (overall) CC line population (Figure 1A) and for males and females, as shown in Figure 1B,C, respectively. The mean body weight change for the entire population of CC lines (*n* = 525/55 CC lines) was observed in varying degrees, with the highest mean values in IL2131, and the mean values from IL2668 representing the lowest value in the overall population. In the male‐only group, the mean body weight change ranged from the highest mean values in IL2131, with the mean values from IL2462 representing the lowest value. The mean body weight change ranged from the highest mean values in IL2146, with the mean values from IL2668 representing the lowest in the female‐only group.

### 3.2 Identification of candidate genes within the mapped QTL intervals

A total of 62 QTLs were identified in our study and designated as ObSL (Obesity‐**s**pecific **l**oci) and numbered. The mapped QTLs and their genomic interval locations, peak interval sizes, chromosome specificity, and specific traits are listed in Tables 1, 2, 3, and Figure S1A–C, respectively, representing the overall population, males, and females separately. A total of 28 QTLs were not previously mapped. The genomic intervals of the remaining 34 QTLs were previously reported, and their genomic intervals were fine‐mapped in our study, as shown in Table S2. The genomic positions of 42 of the mapped QTLs overlapped (clustered) on seven different peaks or close genomic positions, as shown in Table S3.

**TABLE 1:** Summary of significant ObSL quantitative trait locus (QTL) for time series data of body weight traits at different time points of the mice age (following 12 weeks of high‐fat diet [HFD]) for the overall population.

Trait	Chromosome	QTL	Peak (Mb)	CI 50% Size (Mb) (genes)	CI 90% Size (Mb) (genes)	CI 95% Size (Mb) (genes)
ΔBW0‐2	chr14	ObSL1*	57.24	56.57–57.51 0.94 (32)	53.94–59.34 5.40 (267)	52.87–61.00 8.12 (414)
	chr15	ObSL2**	28.45	27.83–29.88 2.05 (8)	24.68–35.34 10.67 (104)	23.64–37.32 13.68 (145)
	chr15	ObSL3*	54.46	53.38–55.65 2.27 (31)	46.50–59.75 13.25 (123)	44.88–62.17 17.29 (158)
ΔBW0‐4	chr11	ObSL4**	19.05	18.39–19.27 0.88 (15)	16.81–20.29 3.48 (52)	15.84–20.80 4.96 (72)
	chr16	ObSL5**	49.36	47.87–50.47 2.60 (37)	42.65–56.35 13.69 (176)	40.98–58.17 17.19 (211)
	chr16	ObSL6**	75.55	73.73–77.92 4.19 (39)	66.54–84.62 18.09 (121)	65.73–85.32 19.58 (140)
ΔBW0‐8	chr5	ObSL7**	58.57	57.18–60.17 2.99 (14)	53.02–65.27 12.25 (86)	51.48–67.14 15.65 (147)
	chr15	ObSL8**	28.45	28.17–29.05 0.87 (2)	25.69–31.48 5.79 (51)	24.83–32.46 7.63 (68)
ΔBW0‐10	chr5	ObSL9**	50.85	49.38–52.80 3.42 (26)	44.19–57.56 13.38 (98)	42.17–59.08 16.91 (129)
	chr5	ObSL10**	56.40	55.26–57.88 2.62 (15)	51.82–62.10 10.28 (64)	50.27–64.20 13.93 (88)
ΔBW0‐12	chr1	ObSL11*	18.08	17.88–18.61 0.73 (8)	15.77–20.78 5.01 (45)	14.54–21.74 7.20 (76)
	chr5	ObSL12**	53.33	50.67–55.07 4.39 (38)	45.30–60.96 15.67 (103)	44.11–62.51 18.40 (117)
	chr11	ObSL13**	18.24	17.68–18.46 0.78 (7)	15.58–19.44 3.86 (48)	14.71–20.08 5.37 (61)
ΔBW2‐4	chr2	ObSL14*	118.08	117.48–118.53 1.05 (17)	113.41–121.34 7.94 (178)	111.83–122.67 10.84 (273)
	chr16	ObSL15*	49.61	45.39–51.30 5.91 (82)	40.14–57.93 17.79 (213)	39.69–59.10 19.41 (248)
	chr16	ObSL16**	75.44	74.37–77.67 3.30 (32)	68.53–83.95 15.42 (105)	66.36–84.97 18.61 (132)
ΔBW6‐8	chr1	ObSL17**	62.39	61.92–62.81 0.89 (7)	60.14–64.32 4.18 (72)	59.13–64.85 5.73 (106)
	chr1	ObSL18**	32.96	31.00–33.84 2.84 (32)	28.28–36.11 7.83 (86)	26.82–37.24 10.42 (120)
	chr12	ObSL19**	29.26	26.12–30.00 3.88 (32)	19.97–37.75 17.78 (236)	19.26–38.85 19.59 (255)
	chr19	ObSL20*	54.50	52.47–55.21 2.75 (30)	45.61–58.78 13.17 (170)	44.80–60.49 15.68 (229)
ΔBW8‐10	chr10	ObSL21*	124.66	124.34–124.99 0.65 (2)	123.11–126.16 3.05 (14)	122.25–126.67 4.41 (25)
ΔBW10‐12	chr13	ObSL22*	113.98	112.60–115.71 3.11 (54)	107.42–120.23 12.81 (193)	105.81–122.14 16.33 (217)
ΔBW6‐12	chr1	ObSL23*	58.57	58.16–59.24 1.08 (30)	56.33–62.25 5.92 (108)	53.66–63.28 9.62 (165)
	chr1	ObSL24**	40.01	39.47–40.58 1.11 (24)	35.90–43.34 7.45 (126)	33.90–45.91 12.01 (198)
	chr2	ObSL25*	57.50	56.75–58.59 1.85 (29)	54.14–61.94 7.80 (95)	52.76–64.45 11.69 (127)
	chr5	ObSL26**	59.83	57.27–62.83 5.56 (24)	51.48–69.04 17.56 (165)	50.35–69.67 19.31 (177)
	chr6	ObSL27**	94.57	93.03–98.54 5.51 (59)	86.98–103.97 16.98 (243)	85.56–104.52 18.97 (288)
	chr12	ObSL28*	10.47	7.94–12.95 5.01 (56)	2.18–19.13 16.95 (198)	1.27–20.40 19.13 (218)

*Note*: The levels of genome‐wide significance thresholds were **95% and *90%.

### 3.3 Mapping new QTL

Our results showed that 28 new QTLs were not mapped in previous reports associated with our phenotypic data. The genomic interval positions of 16 of these QTLs overlapped with previously mapped QTLs (Tables S2 and S3), whereas 12 are unique. The first new QTL ObSL1 mapped during our study was on chromosome 14 at 52.87–61.00 Mbp, peaking at 57.24 Mbp in the overall population at BW02. The other observed new QTL of ΔBW02 in the overall population were *ObSL*2 and *ObSL*3, both located on chromosome 15, with interval positions of 23.64–37.32 and 44.88–62.17 Mbp, respectively, and their respective peak positions at 28.45 and 54.46 Mbp. In ΔBW04 phenotype in the overall population, three new QTLs, ObSL4, ObSL5, and ObSL6, were identified, the former on chromosome 11 and the later two on chromosome 16. In ΔBW08 phenotype in the overall population, a single new QTL ObSL8 was identified on chromosome 15 with an interval position of 24.83–32.46 Mbp with its respective peak positions at 28.45 Mbp. In ΔBW012 phenotype in the overall population, a single new QTL ObSL13 was identified on chromosome 11 with an interval position of 14.71–20.08 Mbp, reaching its peak position at 18.24 Mbp. In the overall population with phenotype ∆BW2‐4, three new QTLs, ObSL14, ObSL15, and ObSL16, were identified on two different chromosomes with their respective interval positions of 118.83–122.67, 39.69–59.10, and 66.36–84.97 Mbp. In the overall population with phenotypes of ∆BW810 and ∆BW1012, two new single QTLs, ObSL21 and ObSL22, were identified, respectively, on chromosomes 10 and 13 in each phenotype. In the overall population with phenotype ∆BW612, a single new QTL ObSL27 was identified on chromosome 6 with an interval position of 85.56–104.52 Mbp and a peak position at 94.57 Mbp.

The other major group of mapped QTLs was from the male population. Here, in males on chromosome 3, the identified QTL at ΔBW02 was ObSL29 on an interval position of 77.92–89.10 Mbp, with its peak position at 81.65 Mbp. In ΔBW04, two new QTLs, ObSL39 and ObSL40, were identified on chromosomes 11 and 15, respectively, with the respective interval positions of 16.92–20.24 and 24.70–31.64 Mbp. For ΔBW06 phenotype, a new QTL ObSL41, with the interval position of 24.86–31.75 Mbp, peaked at 28.04 Mbp was identified on chromosome 15. With the ΔBW08 phenotype, a novel QTL, ObSL43, with its respective interval position of 25.38–30.97 Mbp, was identified on chromosome 15. In males with phenotype ∆BW24, two new QTLs, ObSL45 and ObSL46, were identified on chromosome 2 with the respective interval positions of 100.99–117.72 Mbp, reaching their peaks at 109.23 and 115.19 Mbp. In males with ∆BW10–12 phenotype, a new QTL ObSL51 was identified on chromosome 4 with the interval position of 95.56–114.02 Mbp, reaching its peak at 104.57. In females with phenotypes of ΔBW08 and ∆BW68, two new QTLs, ObSL56 and ObSL60, were identified, respectively, on chromosomes 16 and 13, with the respective interval positions of 44.01–56.03 and 32.12–46.94 Mbp, reaching their peaks at 49.24 and 8.75 Mbp.

### 3.4 Fine‐mapping of previously identified QTL

The other 34 QTLs observed during our study were also new, but they had already been identified by earlier researchers, although our study fine‐mapped their interval position. In the overall population, 14 fine‐mapped QTLs were ObSL7, ObSL9, ObSL10, ObSL11, ObSL12, ObSL17, ObSL18, ObSL19, ObSL20, ObSL23, ObSL24, ObSL25, ObSL26, and ObSL28 on different chromosomes (Table 2). Similarly, in the males‐only group, 12 fine‐mapped QTL were observed on five different chromosomes throughout the genome (Table 2). In the female population, eight fine‐mapped QTLs were observed: ObSL53, ObSL54, ObSL55, ObSL57, ObSL58, ObSL59, ObSL61, and ObSL62, located on chromosomes 1, 3, and 13 (Table 2). Regarding the genome‐wide significance threshold, three sets of QTLs were identified during our study (Figure S1A–C). The first group had a 50% genome‐wide significance threshold. The second group had a 90% genome‐wide significance threshold, whereas the third group had a 95% genome‐wide significance threshold.

**TABLE 2:** Summary of significant ObSL quantitative trait locus (QTL) for time series data of body weight traits at different time points of the mice age (following 12 weeks of high‐fat diet [HFD]) for males only.

Interval	Chromosome	QTL	Peak (Mb)	50% CI Size (Mb) (genes)	CI 90% Size (Mb) (genes)	CI 95% Size (Mb) (genes)
ΔBW0‐2	chr3	ObSL29*	81.65	81.28–83.74 2.46 (26)	79.16–87.90 8.73 (125)	77.92–89.51 11.59 (246)
	chr5	ObSL30**	23.91	21.91–25.59 3.67 (105)	16.89–31.48 14.59 (288)	15.27–32.98 17.70 (343)
	chr5	ObSL31**	55.18	52.53–58.90 6.37 (46)	47.30 64.62 17.32 (114)	45.93–65.12 19.18 (126)
	chr7	ObSL32**	47.05	46.85–47.16 0.31 (11)	45.56–48.17 2.60 (106)	44.67–50.48 5.81 (213)
	chr7	ObSL33*	34.19	33.94–34.61 0.67 (27)	32.63–35.98 3.34 (100)	31.32–37.86 6.54 (142)
	chr11	ObSL34*	18.26	17.71–18.51 0.80 (9)	16.45–19.49 3.04 (45)	15.90–20.02 4.12 (54)
	chr15	ObSL35**	28.44	28.10–29.03 0.93 (3)	25.33–31.74 6.40 (59)	24.20–34.43 10.22 (91)
	chr15	ObSL36**	58.94	58.27–59.36 1.09 (23)	55.45–61.84 6.38 (83)	53.13–62.98 9.85 (118)
ΔBW0‐4	chr5	ObSL37**	25.20	23.24–26.67 3.43 (104)	17.78–32.51 14.72 (308)	16.26–33.80 17.53 (351)
	chr5	ObSL38**	56.60	55.07–58.23 3.16 (16)	49.46–63.49 14.03 (80)	47.99–65.35 17.36 (127)
	chr11	ObSL39**	18.84	18.44–19.04 0.59 (13)	17.44–19.83 2.38 (30)	16.92–20.24 3.32 (50)
	chr15	ObSL40**	28.04	27.83–28.40 0.56 (6)	26.24–30.64 4.40 (37)	24.70–31.64 6.93 (63)
ΔBW0‐6	chr15	ObSL41**	28.04	27.76–28.55 0.79 (6)	25.92–30.88 4.96 (39)	24.86–31.75 6.88 (63)
ΔBW0‐8	chr5	ObSL42**	56.27	54.90–57.73 2.83 (15)	50.20–62.20 12.00 (71)	48.46–64.05 15.58 (94)
	chr15	ObSL43**	28.04	27.83–28.39 0.55 (6)	26.23–30.14 3.90 (32)	25.38–30.97 5.58 (45)
ΔBW0‐12	chr5	ObSL44**	53.20	51.08–55.46 4.37 (37)	45.78–61.13 15.35 (92)	44.01–62.36 18.35 (120)
ΔBW2‐4	chr2	ObSL45**	109.23	106.98–110.45 3.47 (37)	102.44–116.66 14.21 (271)	100.99–117.72 16.73 (304)
	chr2	ObSL46**	115.19	113.58–116.57 2.99 (35)	106.13–121.65 15.52 (332)	105.48–123.63 18.15 (393)
ΔBW6‐8	chr1	ObSL47**	62.67	62.24–62.97 0.73 (8)	60.90–64.07 3.17 (50)	60.18–64.38 4.19 (71)
	chr7	ObSL48*	58.64	57.26–60.95 3.69 (241)	50.55–67.88 17.32 (404)	49.49–68.41 18.91 (419)
	chr12	ObSL49**	29.17	26.78–29.87 3.09 (24)	20.84–36.57 15.73 (218)	19.76–38.17 18.41 (240)
ΔBW8‐10	chr5	ObSL50**	76.80	75.44–78.47 3.02 (46)	69.82–83.92 14.10 (152)	68.07–85.42 17.34 (168)
ΔBW10‐12	chr4	ObSL51**	104.57	102.78–106.55 3.76 (51)	97.03–113.02 15.98 (297)	95.56–114.02 18.46 (322)
ΔBW6‐12	chr5	ObSL52**	76.65	73.14–78.54 5.40 (88)	67.44–85.33 17.89 (180)	66.81–86.38 19.57 (201)

*Note*: The levels of genome‐wide significance thresholds were **95% and *90%.

### 3.5 Founder effect

The results of this analysis are presented in Figure S2A–C, respectively, for the overall population, males only, and females only. We evaluated the effects of each founder genotype on the overall population, males, and females ΔBW mapped QTL interval, and estimated the assessed trait. The wild‐derived strains, primarily PWK, were a significant factor in the rise in ΔBW values, as demonstrated by the loci complicated pattern of founder haplotype effects, except in females where NOD plays the same role instead. Although other strains also contributed (positively or negatively) to the overall QTL effect, the NZO and NOD genotypes decreased this characteristic.

### 3.6 Gene prioritization and functional analysis

The results of the overall population are presented in Table 1 and Figure S1A.

For the DBW0‐2 phenotype, three QTLs (ObSL1, ObSL2, ObSL3), one on chromosome 14 and two on chromosome 15, containing 414, 145, and 158 genes in the 95% CI, respectively, were reported.

Interestingly, one of the systemic processes highly enriched in the MGI mammalian phenotype for this early time point is abnormal prenatal growth/weight/body size and from gene ontology lipid storage. Exostosin glycosyl transferase 1 (*Ext1*) is the highest prioritized gene. *Ext1* is downregulated by a HFD, and this downregulation is considered to exacerbate endoplasmic reticulum (ER) stress and nonalcoholic fatty liver disease (NAFLD) progression.^8^ Other prioritized genes include fat storage‐inducing transmembrane protein 1 (*Fitm1*) and hyaluronan synthase 2 (*Has2*). *Has2* overexpression in mice has been shown to reduce fat accumulation and improve glucose tolerance.^9^

For the DBW6‐12 phenotype, six QTLs (ObSL23, ObSL24, ObSL25, ObSL26, ObSL27, ObSL28), two on chromosome 1, one on chromosome 2, one on chromosome 5, one on chromosome 6, and one on chromosome 12, containing 165, 198, 127, 177, 288, and 218 genes in the 95% CI, respectively, were reported. Enriched systemic processes include increased body size and abnormal pituitary hormone level with notable genes prioritized being gonadotropin‐releasing hormone receptor (*Gnrhr*), ATPase, aminophospholipid transporter (*APLT*), class I, type 8A, member 1 (*Atp8a*1), phosphoribosyl pyrophosphate amidotransferase (*Ppat*), and neuromedin U (*NmU*). *Nmu* ^−/−^ mice are more susceptible to obesity,^10^ and homozygous carriers of *NMU* gene mutations have increased the prevalence of overweight and obesity.^11^

The results for the male population are presented in Table 2 and Figure S1B. For the DBW0‐2 phenotype, eight QTLs (ObSL29, ObSL30, ObSL31, ObSL32, ObSL33, ObSL34, ObSL35, ObSL36), one on chromosome 3, two on chromosome 5, two on chromosome 7, one on chromosome 11, and two on chromosome 15, containing 246, 343, 126, 126, 213, 142, 54, 91, and 118 genes in the 95% CI, respectively, were reported. The enriched systemic processes include impaired glucose tolerance and abnormal adipose tissue physiology and involved several highly prioritized genes shared with the overall population QTL, such as Ppargc1a.

The results of the female population are presented in Table 3 and Figure S1C. For female mice, we observed QTL on chromosome 3 for phenotypes DBW0‐4, DBW0‐6, DBW0‐8, DBW0‐10, and DBW0‐12 (ObSL53, ObSL54, ObSL55, ObSL57, ObSL58), showing strong involvement of the locus in many different time points. ObSL58 has been previously described by our laboratory as ObFL in Binenbaum et al.^12^

**TABLE 3:** Summary of significant ObSL quantitative trait locus (QTL) for time series data of body weight traits at different time points of the mice age (following 12 weeks of high‐fat diet [HFD]) for females only.

Interval	Chromosome	QTL	Peak (Mb)	50% CI Size (Mb) (genes)	CI 90% Size (Mb) (genes)	CI 95% Size (Mb) (genes)
ΔBW0‐4	chr3	ObSL53**	132.27	132.01–132.62 0.60 (3)	131.01–133.50 2.48 (32)	130.39–133.99 3.60 (43)
ΔBW0‐6	chr3	ObSL54**	132.34	132.15–132.61 0.46 (2)	131.39–133.26 1.87 (20)	130.98–133.58 2.60 (33)
ΔBW0‐8	chr3	ObSL55*	132.29	132.11–132.65 0.53 (3)	131.19–133.55 2.35 (29)	130.81–133.85 3.04 (39)
	chr16	ObSL56**	49.24	48.53–50.13 1.60 (25)	45.27–53.50 8.23 (95)	44.01–56.03 12.02 (147)
ΔBW0‐10	chr3	ObSL57**	132.34	132.14–132.60 0.45 (2)	131.26–133.22 1.95 (25)	130.98–133.58 2.59 (33)
ΔBW0‐12	chr3	ObSL58**	132.29	131.91–132.63 0.72 (5)	130.56–133.58 3.01 (41)	129.67–134.08 4.40 (58)
ΔBW6‐8	chr1	ObSL59*	32.95	32.42–33.76 1.33 (19)	29.94–35.96 6.02 (72)	28.86–37.32 8.45 (112)
	chr13	ObSL60**	8.75	8.48–9.01 0.52 (11)	7.19–9.91 2.71 (29)	6.58–10.28 3.70 (34)
ΔBW6‐12	chr1	ObSL61**	38.18	37.62–39.54 1.92 (40)	34.09–43.66 9.57 (154)	32.12–46.94 14.82 (230)
	chr18	ObSL62**	90.42	88.98–90.59 1.60 (17)	85.98–91.67 5.68 (31)	84.36–92.34 7.95 (49)

*Note*: The levels of genome‐wide significance thresholds were **95% and *90%.

## 4 DISCUSSION

To better comprehend the variations in body weight between the many CC lines exhibiting genetic diversity,^12^, ^13^, ^14^ we looked into the cross‐population between several CC mice lines. These lines are not just closely linked genetically, but they also share known obesity loci on separate chromosomes, which accounts for the total variation in obesity across all these lines.^15^ Using QTL mapping to analyze the time series body weight data, we could pinpoint distinct body weight QTL on various chromosomes that explains population variance across varying periods, as reported previously.^15^, ^16^

Based on our previous studies and reports, as presented here, we have demonstrated that male and female mice respond differently to the development of obesity when maintained on a HFD. It was speculated that different genes could be involved in this variation. Indeed, our QTL mapping results have shown that male and female mice have different QTLs associated with obesity development. We leveraged the large, well‐studied cohort, enabling us to perform combined analyses for both males and females, as well as separate analyses for male and female mice. Given the large number of mice used, it became evident to use a multipronged approach for this study.

In response to the environmental and nutritional challenge of a HFD (42%), this work employs a novel CC mouse model to investigate the intricate genetic architecture of obesity. Additional research is necessary to fully comprehend the biological functions and mechanisms of the obesity‐associated loci thus far in human GWAS surveys.^17^, ^18^, ^19^ Several genes inside of our QTL intervals have not been associated with obesity in humans. As a result of finding new genes linked to the feature under investigation, our findings are novel. Finding phenotype–genotype connections has been successful for recent human GWAS. Numerous benefits, particularly for GWAS studies, make mouse models desirable. These benefits include the availability of genetic experimental tools, mouse genomic data resources, ease of breeding, and the capacity to provide highly controlled environmental conditions.^20^ CC mouse populations that resemble models of various human groups were identified to analyze phenotypic–genotypic relationships and highlight interpopulation heterogeneity regarding similar environmental exposures.

After the QTL is mapped, basic and clinical researchers will use it extensively. Subsequent steps include candidate gene search and validation, translation of functional signaling pathways to high‐level human genetics, and candidate gene translation. The top candidate genes for the novel QTL on chromosome 15 associated with body weight are *Gpt*, *Cbx*6, *Apol*6, and *Apol*8. *Gpt* is an essential gene that codes for alanine aminotransferase 1 (*ALT*1), facilitating amino acid transamination into gluconeogenesis and the urea cycle. For the QTL on chromosome 16, *Trap*1 (TNF receptor‐associated protein 1) and *Rrn3* (RNA polymerase I transcription factor homolog) were ranked as the top two candidate genes, which have been previously described in Delpero et al.,^16^ to be associated with final body weight at week 20.

Many factors, including environmental and genetic factors, influence body weight. Although some genes are known for their significant influence on body weight when dysfunctional (e.g., leptin, leptin receptor, *MC*4*R*, *POMC*), most genes have a small impact, and many more are still undiscovered.^2^ Genes with minor effects are more complex to track, especially when their effect is time dependent, so they mainly act during specific periods, such as puberty. Although many environmental factors are well known, the contribution of many genetic factors and the interaction between genetic determinants and the environment are still unknown.^21^ QTL mapping under different conditions, such as time series data, genetic background, and dietary condition, and the subsequent identification of genomic regions and candidate genes influencing obesity in mice and humans are essential to help understand the genetic contribution and its interaction with environmental factors in this common complex human disease. Finally, in our study, over 62 new QTLs were identified, which were thoroughly researched in the literature. Among these, 28 QTLs associated with defined traits were observed and found not to be reported previously, whereas an additional 34 QTLs were fine‐mapped.

It is often recognized that when it comes to obesity, genotypes have a significant impact on phenotypes. The findings of this study provide more evidence that phenotypes are influenced by genetics. Overall obesity is influenced by a number of hereditary factors, and the current study's findings are in line with earlier research in humans and mice.^22^ Moreover, as highlighted in a recent review on leveraging mouse resources for investigating complex characteristics, available data in the eight founder strains investigated here support heritability of body weight. Our findings show how body weight and other particular parameters are distributed and heritable. The eight founder strains of the CC and diversity outbred mouse populations differed significantly for each parameter, and we also observed considerable heritability. After adjusting for body weight, most heritability estimates stayed quite high, indicating the role of genetics in obesity.

## AUTHOR CONTRIBUTIONS

**Hanifa J. Abu‐Toamih Atamni:** Conceptualization; data curation; formal analysis. **Iqbal M. Lone:** Data curation; formal analysis; methodology; writing – original draft. **Ilona Binenbaum:** Data curation; formal analysis; methodology; software; writing – original draft. **Kareem Midlej:** Data curation; formal analysis. **Eleftherios Pilalis:** Data curation; formal analysis; supervision. **Richard Mott:** Conceptualization; data curation; formal analysis; funding acquisition; resources; writing – original draft. **Aristotelis Chatziioannou:** Data curation; formal analysis; software; validation; writing – original draft. **Fuad A. Iraqi:** Conceptualization; data curation; funding acquisition; investigation; methodology; project administration; resources; software; supervision; validation; visualization; writing – original draft; writing – review and editing.

## FUNDING STATEMENT

This work was supported by the Hendrech and Eiran Gotwert Fund for studying diabetes, Wellcome Trust grants 085906/Z/08/Z, 075491/Z/04 and 090532/Z/09/Z, core funding by Tel‐Aviv University (TAU), Israeli Science Foundation (ISF) grants 429/09, 961/15 and 1085/18, United States‐Israel Binational Science Foundation (BSF) grant 2015077, German Israeli Science Foundation (GIF) grant I‐63‐410.20‐2017. Ilona Binenbaum's PhD thesis was supported by a scholarship from the State Scholarship Foundation in Greece (Athens) (Operational Program “Human Resources Development—Education and Lifelong Learning” Partnership Agreement (PA) 2014–2020).

## CONFLICT OF INTEREST STATEMENT

Aristotelis Chatziioannou and Eleftherios Pilalis are cofounders of the e‐NIOS Applications PC. Besides, no other competing financial interests or associations might pose a conflict of interest (e.g., pharmaceutical stock ownership, consultancy). Fuad A. Iraqi is an editorial board member of Animal Models and Experimental Medicine (*AMEM*) and a corresponding author of this article. To minimize bias, he was excluded from all editorial decision making related to the acceptance of this article for publication.

## Supporting information


**Supplementary Figure 1.** Genome scan of quantitative trait loci (QTLs) associated with total time series data of body weight at different time points of the mice age of different collaborative cross (CC) lines in response to high‐fat diet (HFD, 42%) challenge in the overall population (A), males only (B), and females only (C) in a population of 55 CC lines after 12 weeks on high‐fat (42% fat) dietary challenge. The *X*‐axis represents the 19 mouse chromosomes and the position of mapped QTL on the chromosome. The *Y*‐axis represents the logP of the test of association between locus and body weight trait. QTL based on permutation genome‐wide test, a significant level of *p* ≤ 0.05, was identified.
**Supplementary Figure 2**. The estimated effect size on the total time series data of body weight at different time points for the eight collaborative cross (CC) founder strains for different chromosome quantitative trait locus (QTL) in the overall population (A), male population only (B), and female population only (C). The *X*‐axis represents eight founder strains of the CC mice. The *Y*‐axis represents the haplotype effect size of the CC founder at the body weight QTL.

**Supplementary Table 1.** Summary of the used CC lines in our study. The name of each CC line is designated as IL#, which appears under the column CC lines. The number of males and females in each CC line is provided. Abbreviation: CC, collaborative cross.

**Supplementary Table 2.** Summary of significant **Ob**esity‐**S**pecific **L**ocus, ***ObSL*** quantitative trait locus (QTL) for time series data of body weight traits at different time points of the mice age (following 12 weeks of high‐fat diet [HFD]) fine‐mapped by our study. The levels of genome‐wide significance thresholds were **95% and *90%.

**Supplementary Table 3.** Summary of significant **Ob**esity‐**S**pecific **L**ocus, ***ObSL*** quantitative trait locus (QTL) for time series data of body weight traits at different time points of the mice age (following 12 weeks of high‐fat diet [HFD]), with overlapping of the genomic along with their linkage groups on each chromosome indicated by different colors. The levels of genome‐wide significance thresholds were **95% and *90%.

## Data Availability

All CC lines were genotyped with a high‐density custom‐designed Mouse Universal Genotyping Array (MegaMUGA)—built on an Illumina platform consisting of approximately 230 000 SNPs, which are available at http://mtweb.cs.ucl.ac.uk/mus/www/preCC/MEGA_MUGA/Mar2015.MEGA+MDA+MUGA/.^6^ The phenotype data are also available, upon request, from the corresponding author.
