# Supplementary material for: Mapping quantitative trait loci underlying body weight changes that act at different times during high‐fat diet challenge in collaborative cross mice
Source: Animal Model Exp Med. 2026 Mar 6;9(3):621–9. doi: 10.1002/ame2.70144 (PMC13176103; doi:10.1002/ame2.70144)
Supplement: Supplementary file 1 — Supplementary Figure 1. Genome scan of quantitative trait loci (QTLs) associated with total time series data of body weight at different time points of the mice age of different collaborative cross (CC) lines in response to high‐fat diet (HFD, 42%) challenge in the overall population (A), males only (B), and females only (C) in a population of 55 CC lines after 12 weeks on high‐fat (42% fat) dietary challenge. The X‐axis represents the 19 mouse chromosomes and the position of mapped QTL on the chromosome. The Y‐axis represents the logP of the test of association between locus and body weight trait. QTL based on permutation genome‐wide test, a significant level of p ≤ 0.05, was identified. Supplementary Figure 2. The estimated effect size on the total time series data of body weight at different time points for the eight collaborative cross (CC) founder strains for different chromosome quantitative trait locus (QTL) in the overall population (A), male population only (B), and female population only (C). The X‐axis represents eight founder strains of the CC mice. The Y‐axis represents the haplotype effect size of the CC founder at the body weight QTL. [file AME2-9-621-s001.pdf]

Supplementary Figure 1A.

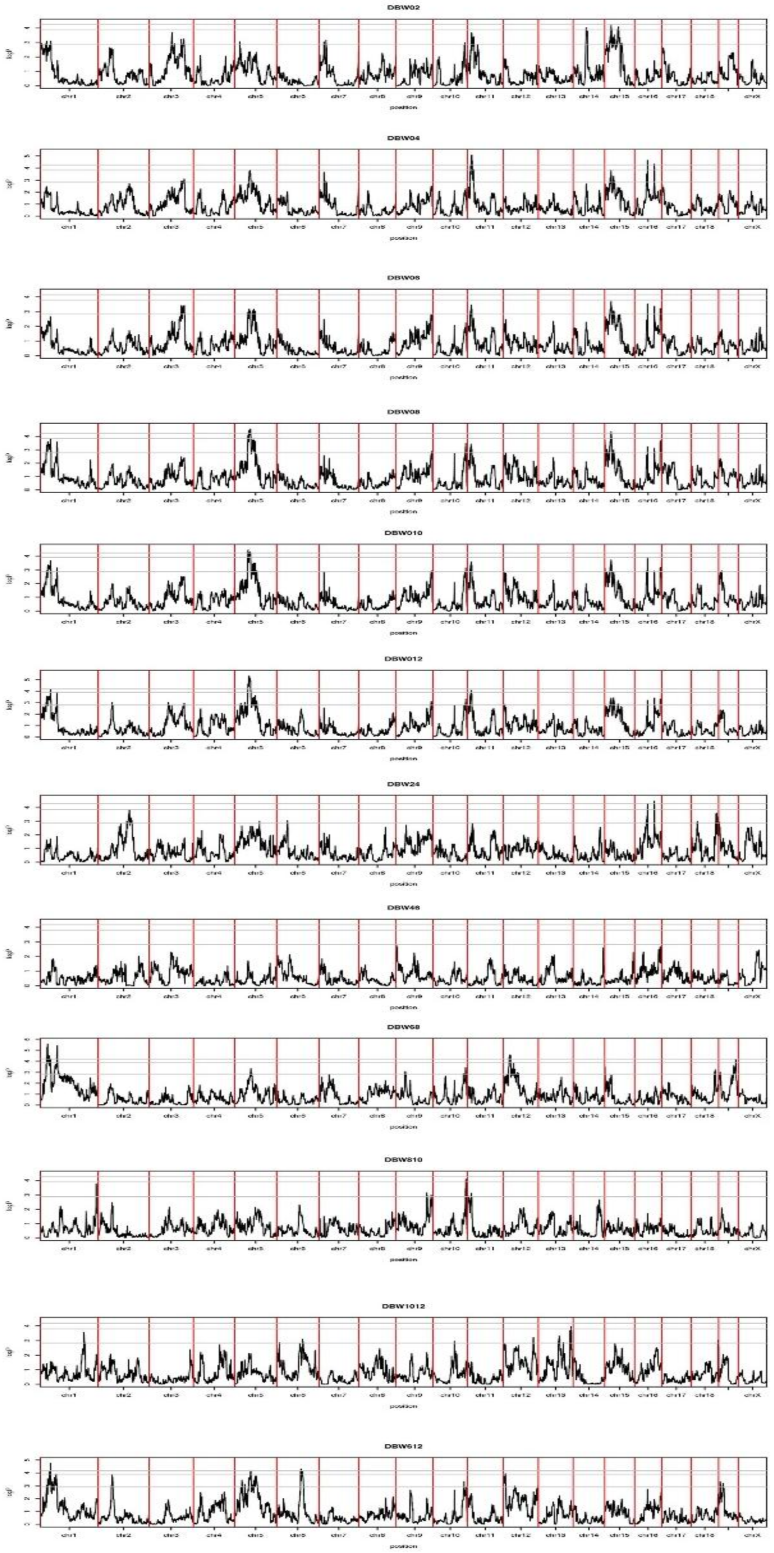

Supplementary Figure 1B.

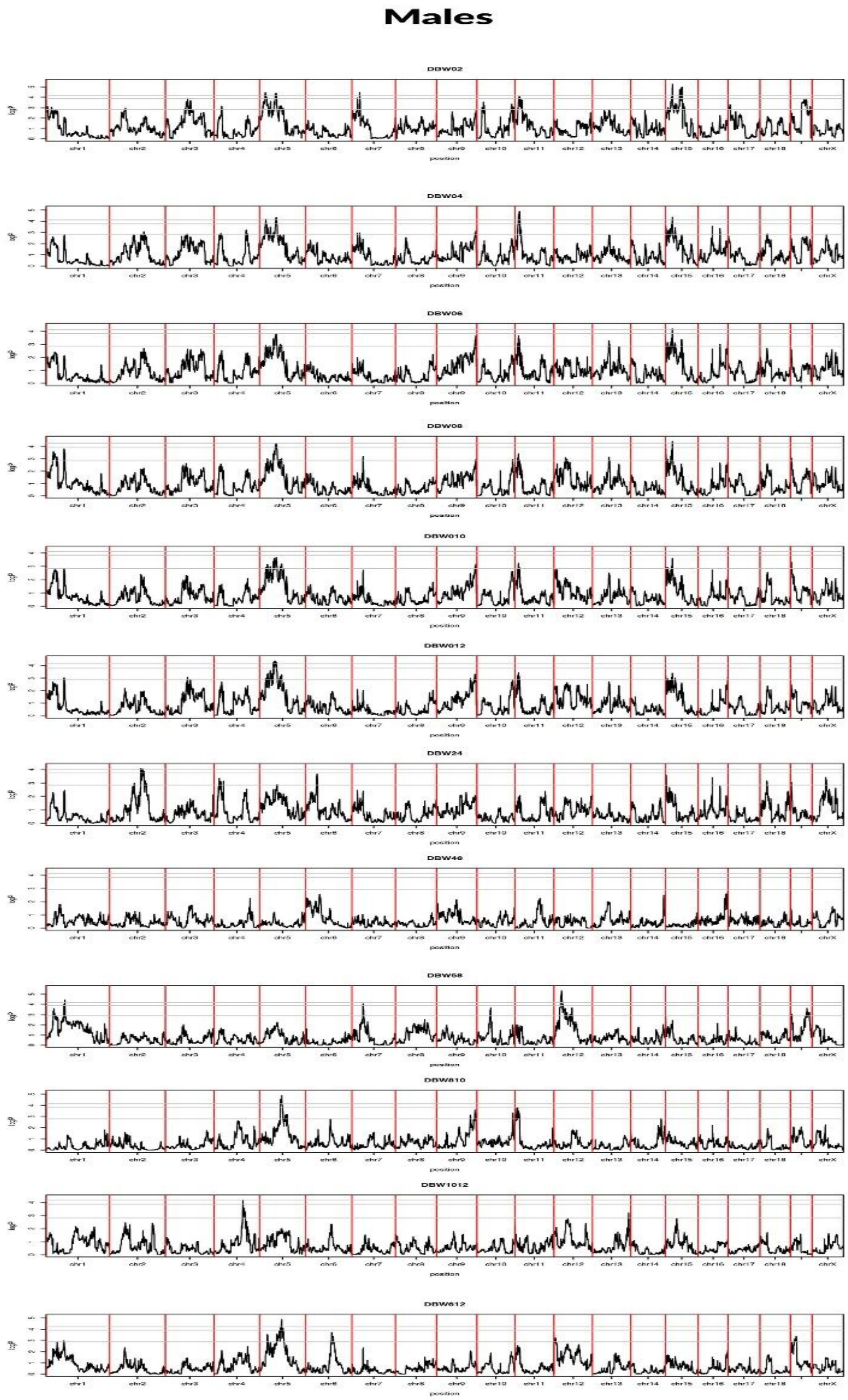

Supplementary Figure 1C.

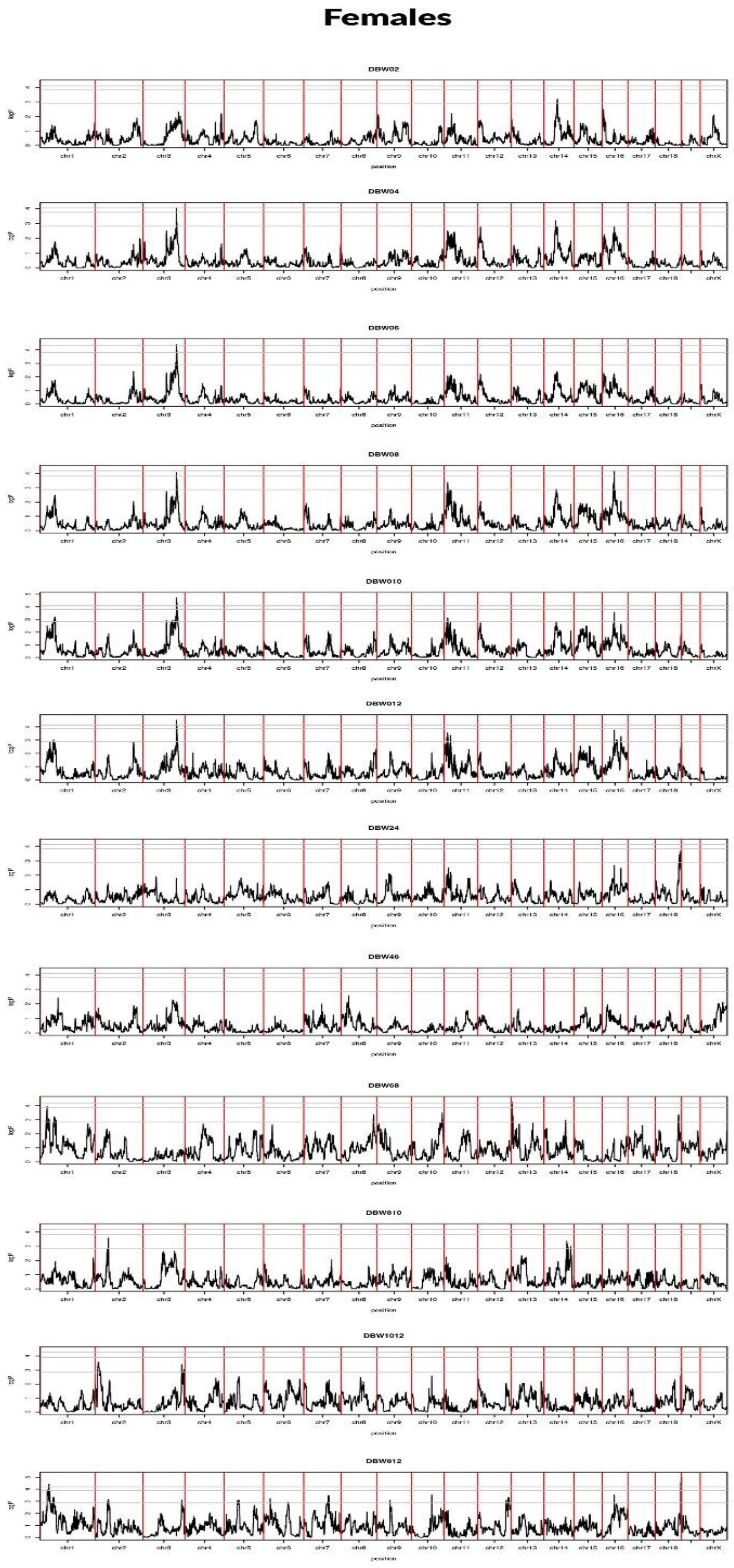

Supplementary Figure 2A.

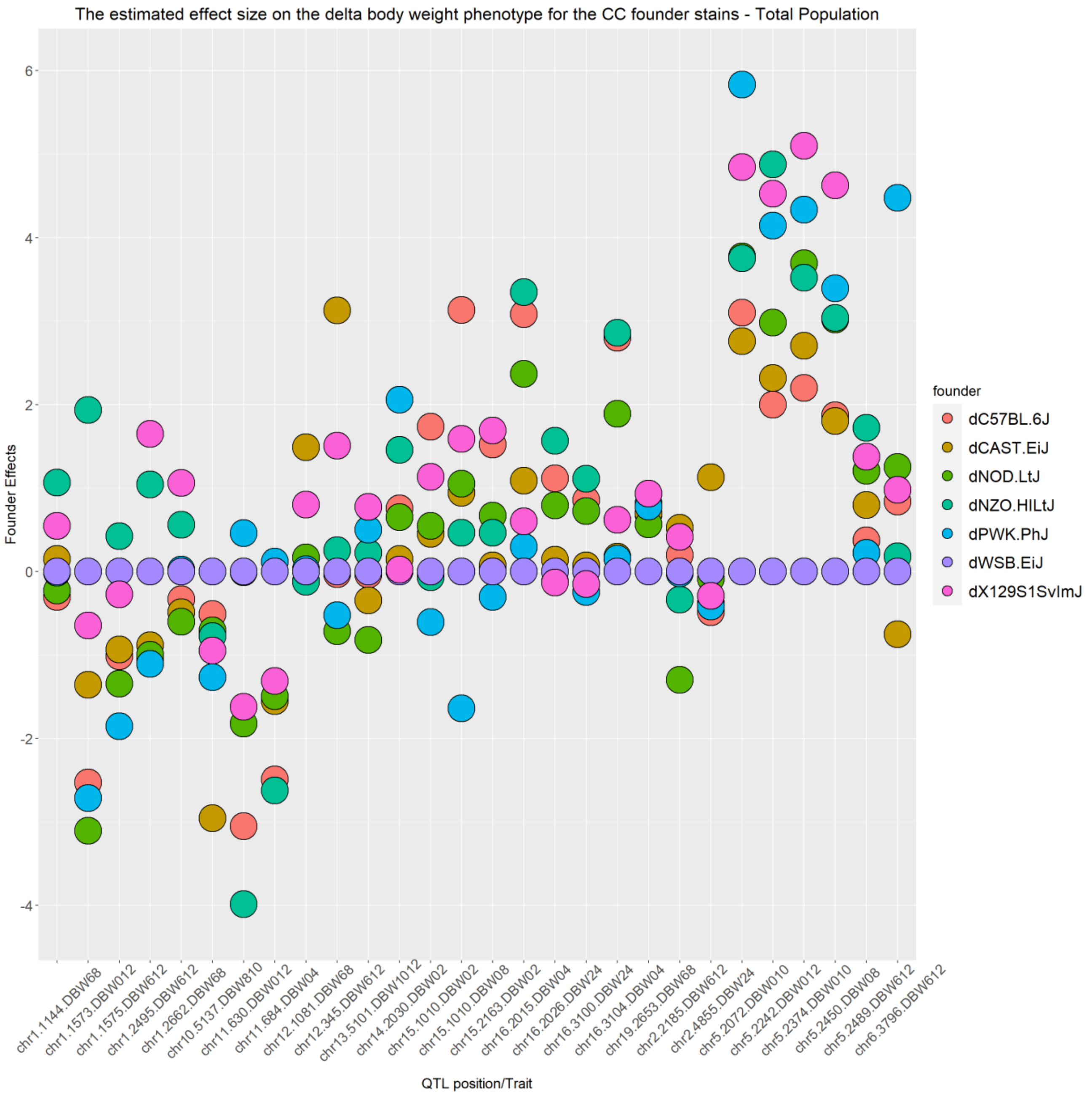

Supplementary Figure 2B.

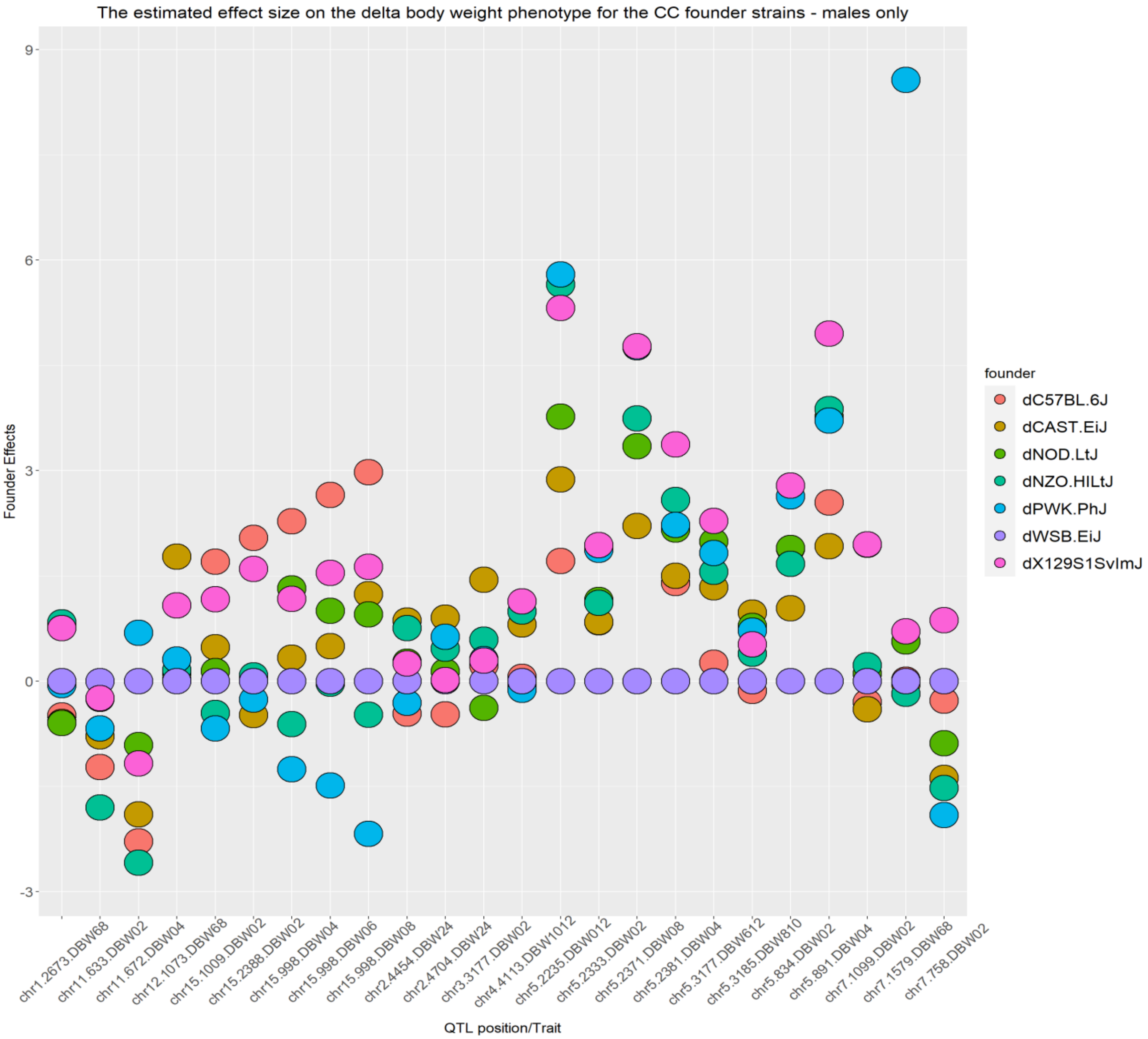

Supplementary Figure 2C.

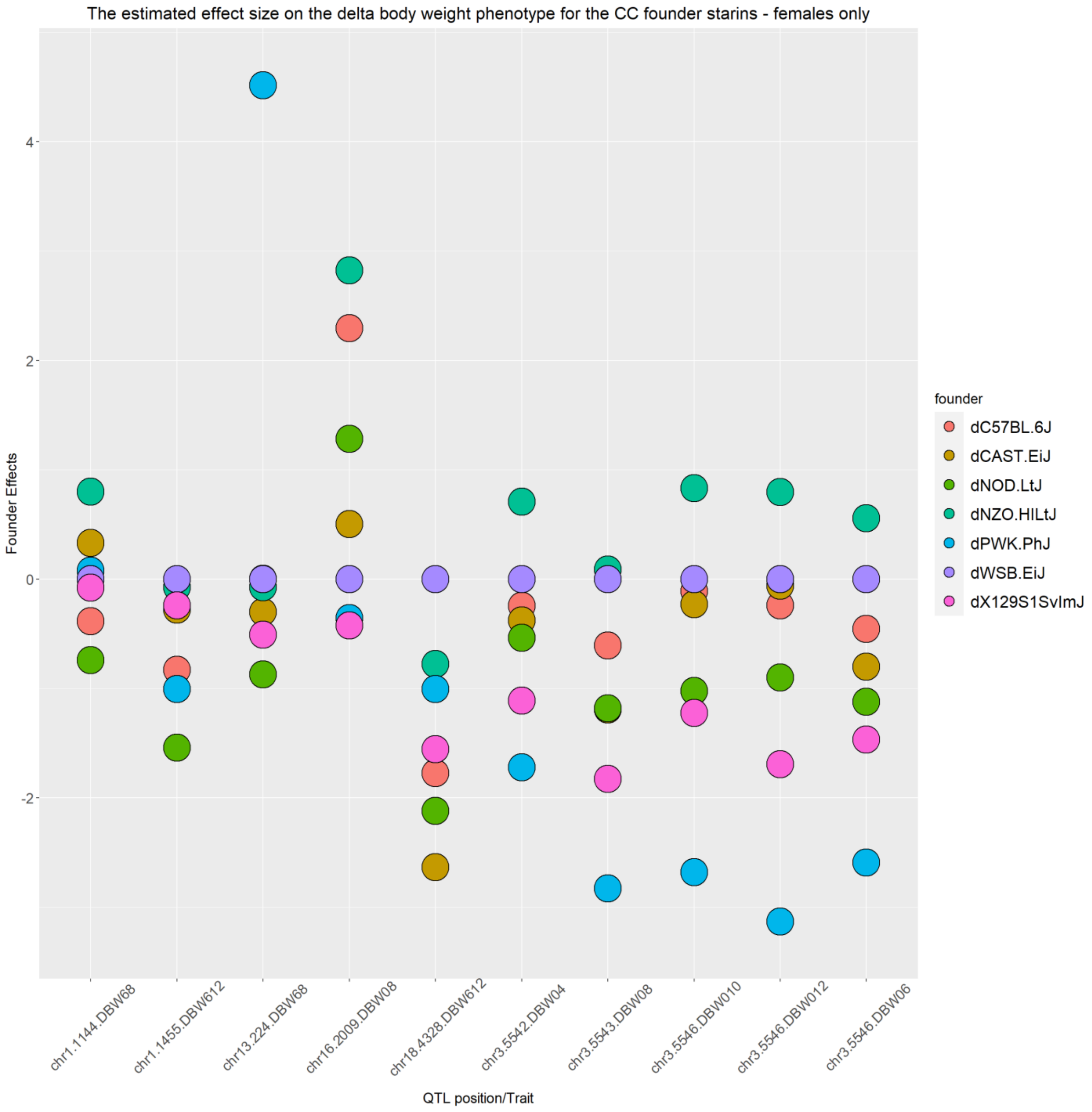

Free access to SNP genotypes of all the CC lines used in this study:

http://mtweb.cs.ucl.ac.uk/mus/www/preCC/
