# Supplementary material for: Mapping quantitative trait loci underlying body weight changes that act at different times during high‐fat diet challenge in collaborative cross mice
Source: Animal Model Exp Med. 2026 Mar 6;9(3):621–9. doi: 10.1002/ame2.70144 (PMC13176103; doi:10.1002/ame2.70144)
Supplement: Supplementary file 2 — Supplementary Table 1. Summary of the used CC lines in our study. The name of each CC line is designated as IL#, which appears under the column CC lines. The number of males and females in each CC line is provided. Abbreviation: CC, collaborative cross. [file AME2-9-621-s003.pdf]

Supplementary Table 1.

CC Line	Female	Male	Total
IL1061	3	9	12
IL111	15	9	24
IL1141	2	5	7
IL1156	1	2	3
IL1379	1	2	3
IL1452	3		3
IL1488	4	2	6
IL1513	4	7	11
IL1675		3	3
IL188	5	10	15
IL1912	7	10	17
IL2011	7	7	14
IL2076	1	1	2
IL2126	9	9	18
IL2131		3	3
IL2146	1	1	2
IL2156	1	16	17
IL219	2	4	6
IL2391	12	1	13
IL2438	3	3	6
IL2457		2	2
IL2460		3	3
IL2462	4	2	6
IL2469		2	2
IL2513	1	1	2
IL2573	3	4	7
IL2668	2	3	5
IL2680	6	5	11
IL2689	7	9	16
IL2693	7	1	8
IL2750	9	11	20
IL3156		5	5
IL3348	4	8	12
IL34	2	2	4
IL3438	5	7	12
IL3480	3	4	7
IL3575	6	5	11
IL3703		3	3
IL3912	7	6	13
IL4052	5	11	16
IL4141	8	5	13
IL4156	6	12	18

IL4348		3	3
IL4438		4	4
IL4457	6	9	15
IL4799	3	7	10
IL519	5	7	12
IL521	8	10	18
IL557	6	4	10
IL611	2	10	12
IL670		4	4
IL68		4	4
IL688		13	13
IL711	10	11	21
IL72	11	7	18
Grand Total	217	308	525
