# Supplementary material for: Mapping quantitative trait loci underlying body weight changes that act at different times during high‐fat diet challenge in collaborative cross mice
Source: Animal Model Exp Med. 2026 Mar 6;9(3):621–9. doi: 10.1002/ame2.70144 (PMC13176103; doi:10.1002/ame2.70144)
Supplement: Supplementary file 3 — Supplementary Table 2. Summary of significant Obesity‐Specific Locus, ObSL quantitative trait locus (QTL) for time series data of body weight traits at different time points of the mice age (following 12 weeks of high‐fat diet [HFD]) fine‐mapped by our study. The levels of genome‐wide significance thresholds were **95% and *90%. [file AME2-9-621-s004.pdf]

Supplementary Table 2.

Trait	Chr	QTL	Peak (Mb)	CI 95% Size (Mb) (Genes)	Previously identified QTL	Genomic interval (Mb)
ΔBW0-8 Overall	Chr5	ObSL7**	58.57	51.48-67.14	*Chol*1/*NZO*	44.00–82.00
ΔDBW0-10 Overall	Chr5	ObSL9**	50.85	42.17-59.08	*Chol*1/*NZO*	44.00–82.00
Overall	Chr5	ObSL10**	56.4	50.27-64.20	*Chol*1/*NZO*	44.00–82.00
ΔBW0-12 Overall	chr5	ObSL12**	53.33	44.11-62.51	*Chol*1/*NZO*	44.00–82.00
ΔBW6-8 Overall	chr1	ObSL17**	62.39	59.13-64.85	Obq8	63.70–85.10
Overall	chr1	ObSL18**	32.96	26.82-37.24	Obq7	24.30–37.90
Overall	chr12	ObSL19**	29.26	19.26-38.85	Fob2	32.10–35.70
Overall	chr19	ObSL20*	54.5	44.80-60.49	*T2dm*2	36.96–59.52
ΔBW6-12 Overall	chr1	ObSL23*	58.57	53.66-63.28	Nob3	40.00–70.00
Overall	chr1	ObSL24**	40.01	33.90-45.91	Obq7	24.30–37.90
Overall	Chr2	ObSL25*	57.5	52.76-64.45	*Nidd*5	57.20-66.60
Overall	Chr5	ObSL26**	59.83	50.35-69.67	*Chol*1/*NZO*	44.00–82.00
Overall		ObSL28*	10.47			

	chr12			1.27-20.40	Bpq17	8.10–52.10
ΔBW0-2 Male	chr5	ObSL30**	23.91	15.27 – 32.98	ObML	25.96 – 36.79
Male	chr5	ObSL31**	55.18	45.93 – 65.12	*Chol*1/*NZO*	44.00–82.00
Male	chr7	ObSL32**	47.05	44.67 – 50.48	Obq15	44.20 – 52.40
ΔBW0-4 Male	chr5	ObSL37**	25.2	16.26 – 33.80	Obq12	21.90–36.10
Male	chr5	ObSL38**	56.6	47.99 – 65.35	*Chol*1/*NZO*	44.00–82.00
ΔBW0-8 Male	chr5	ObSL42**	56.27	48.46 – 64.05	*Chol*1/*NZO*	44.00–82.00
ΔBW0-12 Male	chr5	ObSL44**	53.2	44.01 – 62.36	*Chol*1/*NZO*	44.00–82.00
ΔBW6-8 Male	chr1	ObSL47**	62.67	60.18 – 64.38	Nob3	40.00–70.00
Male	Chr7	ObSL48*	58.64	49.49 – 68.41	Obq15	44.20 – 52.40
Male	chr12	ObSL49**	29.17	19.76 – 38.17	Fob2	32.10–35.70
ΔBW8-10 Male	chr5	ObSL50**	76.8	68.07 – 85.42	*Chol*1/*NZO*	44.00–82.00
ΔBW6-12 Male	chr5	ObSL52**	76.65	66.81 – 86.38	*Chol*1/*NZO*	44.00–82.00
ΔBW6-8 Female	chr1	ObSL59*	32.95	28.86 – 37.32	Obq7	24.30–37.90
ΔBW6-12 Female	chr1	ObSL61**	38.18	32.12 – 46.94	Obq7	24.30–37.90
Female	chr18	ObSL62**	90.42	84.36 – 92.34	*Kcal*1	68.84–90.89
