# Supplementary material for: Mapping quantitative trait loci underlying body weight changes that act at different times during high‐fat diet challenge in collaborative cross mice
Source: Animal Model Exp Med. 2026 Mar 6;9(3):621–9. doi: 10.1002/ame2.70144 (PMC13176103; doi:10.1002/ame2.70144)
Supplement: Supplementary file 4 — Supplementary Table 3. Summary of significant Obesity‐Specific Locus, ObSL quantitative trait locus (QTL) for time series data of body weight traits at different time points of the mice age (following 12 weeks of high‐fat diet [HFD]), with overlapping of the genomic along with their linkage groups on each chromosome indicated by different colors. The levels of genome‐wide significance thresholds were **95% and *90%. [file AME2-9-621-s002.pdf]

Supplementary Table 3.

Trait	Chr	QTL	Peak (Mb)	CI 50% Size (Mb) (Genes)	CI 90% Size (Mb) (Genes)	CI 95% Size (Mb) (Genes)
ΔBW6-8 overall	chr1	ObSL17**	62.39	61.92-62.81 0.89 (7)	60.14-64.32 4.18 (72)	59.13-64.85 5.73 (106)
overall	chr1	ObSL18**	32.96	31.00-33.84 2.84 (32)	28.28-36.11 7.83 (86)	26.82-37.24 10.42 (120)
ΔBW6-12 overall	chr1	ObSL23*	58.57	58.16-59.24 1.08 (30)	56.33-62.25 5.92 (108)	53.66-63.28 9.62 (165)
overall	chr1	ObSL24**	40.01	39.47-40.58 1.11 (24)	35.90-43.34 7.45 (126)	33.90-45.91 12.01 (198)
ΔBW6-8 Male	chr1	ObSL47**	62.67	62.24 – 62.97 0.73 (8)	60.90 – 64.07 3.17 (50)	60.18 – 64.38 4.19 (71)
ΔBW6-8 Female	chr1	ObSL59*	32.95	32.42 – 33.76 1.33 (19)	29.94 – 35.96 6.02 (72)	28.86 – 37.32 8.45 (112)
ΔBW6-12 Female	chr1	ObSL61**	38.18	37.62 – 39.54 1.92 (40)	34.09 – 43.66 9.57 (154)	32.12 – 46.94 14.82 (230)
ΔBW2-4 Male	Chr2	ObSL45**	109.23	106.98 – 110.45 3.47 (37)	102.44 – 116.66 14.21 (271)	100.99 – 117.72 16.73 (304)
Male	Chr2	ObSL46**	115.19	113.58 – 116.57 2.99 (35)	106.13 – 121.65 15.52 (332)	105.48 – 123.63 18.15 (393)
ΔBW0-4 Female	Chr3	ObSL53**	132.27	132.01 – 132.62 0.60 (3)	131.01 – 133.50 2.48 (32)	130.39 – 133.99 3.60 (43)
ΔBW0-6 Female	Chr3	ObSL54**	132.34	132.15 – 132.61 0.46 (2)	131.39 – 133.26 1.87 (20)	130.98 – 133.58 2.60 (33)
ΔBW0-8 Female	Chr3	ObSL55*	132.29	132.11 – 132.65 0.53 (3)	131.19 – 133.55 2.35 (29)	130.81 – 133.85 3.04 (39)
ΔBW0-10 Female	Chr3	ObSL57**	132.34	132.14 – 132.60 0.45 (2)	131.26 – 133.22 1.95 (25)	130.98 – 133.58 2.59 (33)
ΔBW0-12 Female	Chr3	ObSL58**	132.29	131.91 – 132.63 0.72 (5)	130.56 – 133.58 3.01 (41)	129.67 – 134.08 4.40 (58)
ΔBW0-8 overall	Chr5	ObSL7**	58.57	57.18-60.17 2.99 (14)	53.02-65.27 12.25 (86)	51.48-67.14 15.65 (147)
ΔBW0-10 overall	Chr5	ObSL9**	50.85	49.38-52.80 3.42 (26)	44.19-57.56 13.38 (98)	42.17-59.08 16.91 (129)
overall	Chr5	ObSL10**	56.40	55.26-57.88 2.62 (15)	51.82-62.10 10.28 (64)	50.27-64.20 13.93 (88)

ΔBW0-12 overall	chr5	ObSL12**	53.33	50.67-55.07<br>4.39 (38)	45.30-<br>60.96<br>15.67 (103)	44.11-62.51<br>18.40 (117)
ΔBW6-12 overall	Chr5	ObSL26**	59.83	57.27-62.83<br>5.56 (24)	51.48-<br>69.04<br>17.56 (165)	50.35-69.67<br>19.31 (177)
ΔBW0-2 Male	chr5	ObSL30**	23.91	21.91 – 25.59<br>3.67 (105)	16.89 –<br>31.48<br>14.59 (288)	15.27 – 32.98<br>17.70 (343)
Male	chr5	ObSL31**	55.18	52.53 – 58.90<br>6.37 (46)	47.30 –<br>64.62<br>17.32 (114)	45.93 – 65.12<br>19.18 (126)
ΔBW0-4 Male	chr5	ObSL37**	25.20	23.24 – 26.67<br>3.43 (104)	17.78 –<br>32.51<br>14.72 (308)	16.26 – 33.80<br>17.53 (351)
Male	chr5	ObSL38**	56.60	55.07 – 58.23<br>3.16 (16)	49.46 –<br>63.49<br>14.03 (80)	47.99 – 65.35<br>17.36 (127)
ΔBW0-8 Male	chr5	ObSL42**	56.27	54.90 – 57.73<br>2.83 (15)	50.20 –<br>62.20<br>12.00 (71)	48.46 – 64.05<br>15.58 (94)
ΔBW0-12 Male	chr5	ObSL44**	53.20	51.08 – 55.46<br>4.37 (37)	45.78 –<br>61.13<br>15.35 (92)	44.01 – 62.36<br>18.35 (120)
ΔBW8-10 Male	chr5	ObSL50**	76.80	75.44 – 78.47<br>3.02 (46)	69.82 –<br>83.92<br>14.10 (152)	68.07 – 85.42<br>17.34 (168)
ΔBW6-12 Male	chr5	ObSL52**	76.65	73.14 – 78.54<br>5.40 (88)	67.44 –<br>85.33<br>17.89 (180)	66.81 – 86.38<br>19.57 (201)
ΔBW0-4 overall	chr11	ObSL4**	19.05	18.39-19.27<br>0.88 (15)	16.81-<br>20.29<br>3.48 (52)	15.84-20.80<br>4.96 (72)
ΔBW0-12 overall	chr11	ObSL13**	18.24	17.68-18.46<br>0.78 (7)	15.58-<br>19.44<br>3.86 (48)	14.71-20.08<br>5.37 (61)
ΔBW0-2 Male	chr11	ObSL34*	18.26	17.71 – 18.51<br>0.80 (9)	16.45 –<br>19.49<br>3.04 (45)	15.90 – 20.02<br>4.12 (54)
ΔBW0-4 Male	chr11	ObSL39**	18.84	18.44 – 19.04<br>0.59 (13)	17.44 –<br>19.83<br>2.38 (30)	16.92 – 20.24<br>3.32 (50)
ΔBW6-8 overall	chr12	ObSL19**	29.26	26.12-30.00<br>3.88 (32)	19.97-<br>37.75<br>17.78 (236)	19.26-38.85<br>19.59 (255)
ΔBW6-12 overall	chr12	ObSL28*	10.47	7.94-12.95<br>5.01 (56)	2.18-19.13<br>16.95 (198)	1.27-20.40<br>19.13 (218)
ΔBW0-2 Overall	Chr15	ObSL2**	28.45	27.83-29.88<br>2.05 (8)	24.68-<br>35.34<br>10.67 (104)	23.64-37.32<br>13.68 (145)
ΔBW0-8 Overall	chr15	ObSL8**	28.45	28.17-29.05<br>0.87 (2)	25.69-<br>31.48<br>5.79 (51)	24.83-32.46<br>7.63 (68)
ΔBW0-2 Male	chr15	ObSL35**	28.44	28.10 – 29.03<br>0.93 (3)	25.33 –<br>31.74<br>6.40 (59)	24.20 – 34.43<br>10.22 (91)
ΔBW0-4 Male	chr15	ObSL40**	28.04	27.83 – 28.40<br>0.56 (6)	26.24 –<br>30.64<br>4.40 (37)	24.70 – 31.64<br>6.93 (63)

ΔBW0-6 Male	chr15	ObSL41**	28.04	27.76 – 28.55 0.79 (6)	25.92 – 30.88 4.96 (39)	24.86 – 31.75 6.88 (63)
ΔBW0-8 Male	chr15	ObSL43**	28.04	27.83 – 28.39 0.55 (6)	26.23 – 30.14 3.90 (32)	25.38 – 30.97 5.58 (45)
ΔBW0-4 Overall	chr16	ObSL5**	49.36	47.87-50.47 2.60 (37)	42.65-56.35 13.69 (176)	40.98-58.17 17.19 (211)
Overall	chr16	ObSL6**	75.55	73.73-77.92 4.19 (39)	66.54-84.62 18.09 (121)	65.73-85.32 19.58 (140)
ΔBW2-4 Overall	chr16	ObSL15*	49.61	45.39-51.30 5.91 (82)	40.14-57.93 17.79 (213)	39.69-59.10 19.41 (248)
Overall	chr16	ObSL16**	75.44	74.37-77.67 3.30 (32)	68.53-83.95 15.42 (105)	66.36-84.97 18.61 (132)
